# Risk Factors of Thyroid Dysfunction in Patients With Type 2 Diabetes Mellitus

**DOI:** 10.3389/fendo.2019.00440

**Published:** 2019-07-04

**Authors:** Stanley U. Ogbonna, Ignatius U. Ezeani

**Affiliations:** Department of Internal Medicine, Federal Medical Center, Umuahia, Nigeria

**Keywords:** thyroid dysfunction, type 2 diabetes mellitus, hypothyroidism, South East Nigeria, predictors, risk factors

## Abstract

**Background:** Thyroid dysfunction has been widely reported among persons with diabetes (DM) in other parts of the World. In Nigeria, few studies have been reported. This study focused on risk factors for thyroid dysfunction in type 2 diabetes mellitus (T2DM) patients and will therefore add to the Nigerian literature, more so, as it is the first in South-East Nigeria.

**Objective:** To determine the risk factors of thyroid dysfunction in patients with Type 2 DM.

**Methodology:** Three hundred and fifty-four T2DM patients and 118 non-diabetic persons (controls) were recruited for the study. A pretested questionnaire was filled for each subject after due explanations. The subjects were subsequently examined and the findings, including anthropometric values and clinical parameters were documented. Their blood samples were tested for HbA1c, fT3, fT4, and TSH. Information retrieved from patients medical records included: age at diagnosis of DM, duration of DM, complications of DM. The Student's *t*-test, chi square test and regression analysis were used in the analysis of the data obtained. *P* < 0.05 was taken to be statistically significant.

**Results:** About 56.5% of the T2DM patients who participated in this study were females and 62.7% of the controls were females. The T2DM patients had significantly higher BMI than controls (27.6 ± 5.0 kg/m^2^ vs. 26.2 ± 3.8 kg/m^2^, *p* = 0.002). Mean HbA1c was significantly higher in T2DM patients than in the controls (7.8 ± 2.0% vs. 5.8 ± 1.2%, *p* = 0.001). Female gender (OR = 3.8, *p* = 0.002), central obesity (OR = 2.5, 95%CI = 1.5–5.2, *p* = 0.001), DM nephropathy (OR = 4.8, *p* = 0.001), HbA1c ≥7% (OR = 4.3, *p* = 0.025) and duration of DM >5years (OR = 3.3, *p* = 0.012) were significantly associated with thyroid dysfunction in T2DM patients in this study.

**Conclusion:** Female gender, central obesity, DM nephropathy, above normal HbA1c, and duration of DM were risk factors of thyroid dysfunction in type 2 DM patients in this study.

## Introduction

Thyroid dysfunction is a spectrum of disorders of the thyroid gland which manifests either as hyperthyroidism or hypothyroidism and is reflected in the circulating levels of thyroid stimulating hormone (TSH) (1, 2). Thyroid dysfunction may present in one of the following ways- thyroid enlargement (diffuse or nodular); symptoms of thyroid hormone deficiency (hypothyroidism); symptoms of thyroid hormone excess (thyrotoxicosis); some have no symptoms (i.e., the subclinical state) (3). Imbalance in the production of thyroid hormones arises from dysfunction of the thyroid gland itself, the pituitary gland, which produces TSH, or the hypothalamus, which regulates the pituitary gland via Thyrotropin Releasing Hormone (TRH) (4).

Thyroid dysfunctions are common in the general population and it is second only to diabetes as the most common condition to affect the endocrine system. As a result it is common for an individual to be affected by both thyroid diseases and diabetes (5, 6).

Patients with DM are at increased risk of thyroid disease, especially those with poor glycaemic control. The following mechanisms are thought to be responsible. In patients with DM, the nocturnal TSH peak is blunted or abolished; and the TSH response to TRH, from the hypothalamus, is impaired thus leading to hypothyroidism (7). Low T3 levels have been observed in uncontrolled DM. This has been ascribed to the impairment in peripheral conversion of T4 to T3 which normalizes with improvement in glycaemic control (5, 8). This is as a result of the hyperglycaemia-induced reversible reduction of the activities and hepatic concentration of thyroxine 5‘deiodinase (8). Higher levels of circulating insulin associated with insulin resistance has been shown to have a proliferative effect on thyroid tissue resulting in larger thyroid size with increased formation of nodules (5, 9, 10). This may lead to thyroid dysfunction (hyperthyroidism) in patients with type 2 DM.

Metformin decreases thyrotropin levels in patients with hypothyroidism. Prospective and retrospective studies (11) showed that patients with prediabetes and type 2 diabetes mellitus (TDM) had a significantly increased thyroid volume and a higher prevalence of incident goiter and nodules. Furthermore, diabetic patients treated with metformin had a smaller thyroid volume and a lower risk for the formation of thyroid nodules when compared with controls (12, 13). These results suggested that metformin exerts an anti-proliferative activity, providing a rationale for an innovative therapy of thyroid proliferative diseases with metformin.

Metformin is hypothesized to change the affinity and/or quantity of thyroid hormone receptors, increase the central dopaminergic tone or induce activation of the TSH receptor, thus enhancing the effects of thyroid hormones in the pituitary (14).

Other authors suggested that TSH-lowering effect of metformin may be explained by a metformin-induced activation of the adenosine monophosphate-activated protein kinase (AMPK), which is involved in a variety of cellular functions and regulates cellular energy metabolism (15). Indeed, it may be plausible that any central effects of metformin on the TRH/TSH regulation involve the AMPK system. Metformin is proved to have an inhibitory effect on AMPK activity in the hypothalamus where it opposes T 3 (16).

Thyroid hormones affect glucose metabolism through several other mechanisms (17–26). Several studies have reported the prevalence of thyroid dysfunction in diabetic patients but only few studies have assessed the risk factors of thyroid dysfunction in diabetic patients, hence the need for this study. The objective of this study is to determine the risk factors of thyroid dysfunction in patients with Type 2 DM.

## Methodology

The study was conducted at the University of Nigeria Teaching Hospital (UNTH), Enugu, a Federal government tertiary hospital located in Ituku-Ozalla, Enugu state in South-East geographical region of Nigeria. This is a descriptive cross-sectional study involving patients with T2DM attending the Diabetes Clinic, or receiving treatment in the Medical Wards of the UNTH Enugu. Subjects were recruited using systematic sampling. A total number of 360 subjects were recruited from consenting persons with T2DM in the Diabetes Clinic and Medical Wards of the UNTH Enugu. For every three study subjects selected, one consenting person who did not have DM was recruited from the out-patients clinics and other parts of the hospital to serve as the control. Screening for DM was done in controls using the random blood glucose (RBG). Those with RBG of <11.1 mmol/l, had no classic symptoms of hyperglycaemia and were not on any hypoglycemic medications were accepted as controls (27). The inclusion criteria included

- All patients with T2DM irrespective of blood pressure status.
- Patients who have attained 40 years of age at the time of diagnosis of DM (28).
- Those who had no thyroid surgery nor trauma to the neck.
- Subjects with no history of previous exposure to radiation to the neck.
- Those consenting to the study.

Exclusion criteria included:

1. Patients <40 years of age at diagnosis of DM
2. Those with history of neck trauma or surgery
3. Pregnant women
4. Subjects with history of previous exposure of radiation in the neck
5. Non-consenting patients
6. Patients on drugs like amiodarone, lithium, interferon alpha, iodides, beta blockers, carbimazole, propylthiouracil, potassium iodide, lugol's iodine
7. Patients with thyroid disorders
8. Patients previously diagnosed to have T1DM.

### Ethical Clearance

Ethical approval was obtained from the University of Nigeria Health Research Ethics Committee of the UNTH Enugu before commencing the study. Written informed consent was obtained from all subjects participating in the study.

All subjects were interviewed, using a pre-tested structured questionnaire. Demographic information and other relevant data were obtained. The neck was examined for presence of enlarged thyroid gland. Routine examinations for the complications of DM were also carried out. Fundoscopy was done with the assistance of an Ophthalmologist. The participants were examined for peripheral neuropathy using tuning fork (vibration sense) and tendon hammer (deep tendon reflex). Anthropometric measurements such as weight and height were taken.

Anthropometric measurements such as weight and height were taken using a standard scale and stadiometer from Lincoln Mark Medical England; waist and hip circumference were measured using a measuring tape. Body Mass Index (BMI) was calculated from their weights measured in kilograms (kg) and heights in meters (m) using the standard formula (29, 30). Subjects with BMI of between 18 and 25 kg/m^2^ were classified as having normal weight, while those with BMI of 30 kg/m^2^ and above were classified as being obese (31). Waist circumference (WC) was measured in centimeters (cm) along the mid-point between the costal margin and the iliac crest along the mid axillary line (32). The International Diabetes Federation (IDF)'s reference values for male and female were used. In males, WC of ≤94 cm was regarded as normal, while WC of >94 cm was regarded as abnormal. In females, WC of ≤80 cm was regarded as normal while WC of >80 cm was taken to be abnormal (33).

Three consecutive pulse rates were obtained and the mean recorded. Blood pressure (BP) was taken on the right arm with a mercury sphygmomanometer (34). The systolic and diastolic pressures were obtained. The disappearance of the korotkoff's sound (phase V) was the criterion for the diastolic blood pressure, and the average of three consecutive BP readings was recorded. BP was measured in a sitting position after 5 min rest. Normal BP was defined as a systolic BP of <130 mmHg, and or diastolic BP of <80 mmhHg. Those who did not satisfy these criteria were considered to have a high BP, in accordance with the BP target set by the American Diabetes Association (ADA) (35).

All the participants had their blood glucose estimated using the Accu-chek Active glucometer. The patients with type 2 DM had their urine samples tested for presence of urinary protein (albumin) using Combi-3 urinary strips from Medi-Test, Germany. The presence of one + and above of urine albumin was taken to be positive for albuminuria.

Glycated hemoglobin (HbA1c) was estimated using the In-2-it HbA1c device from BIO-RAD Laboratories Flintshire UK. It involves the use of boronate affinity chromatography to separate the glycated fraction from the non-glycated fraction (36). It measures the HbA1c level which reflects the average blood glucose level over the previous 2 or 3 months.

Glycaemic control was assessed with the values of the HbA1c. HbA1c value was used to categorize the DM patients into two groups: good glycaemic control (HbA1c<7%), and poor glycaemic control (HbA1c≥7%) (37).

### Criteria for the Diagnosis of Thyroid Dysfunction

Participants with raised TSH, and low fT3 and fT4 were regarded to have primary hypothyroidism; those that had elevated TSH, but with normal fT3 and fT4 were taken to have subclinical hypothyroidism (38–40). In the same vein, those who had low TSH, and high fT3 and fT4 were regarded to have primary hyperthyroidism; those that had low TSH but with normal fT3, and fT4 were taken to have subclinical hyperthyroidism (39–41). While the subjects with low or normal TSH, but had low fT3 and fT4 were taken to have secondary hypothyroidism (40, 42).

### Procedure for Thyroid Function Assay

Frozen sera from the T2DM subjects and controls were thawed and allowed to attain room temperature. The samples were assayed for free T3, free T4, and TSH, respectively in batches, in three runs, each on a different day. Control samples provided in the reagents kits were analyzed with each run of the analytes following the manufacturer's instructions.

The data generated from the study was analyzed using the Statistical Package for the Social Sciences (SPSS) IBM version 17. A statistical comparison was made with the student *t*-test for quantitative variables like weight, height, blood pressure, serum TSH, serum T3; while Chi-square test was used for comparison of proportions. A *p* < 0.05 was taken as being statistically significant.

## Results

A total of 480 subjects were studied. Complete data for analysis was available for 472 subjects. All the subjects (100%) were of African descent. Three hundred and fifty-four (354) of them were patients with type 2 DM, while 118 subjects who did not have type 2 DM served as the controls.

### Socio-Demographic Characteristics

Females formed the majority of the study population accounting for 56.5% of the type 2 DM patients and 62.7% of the controls. Most of the participants had either tertiary education (38.3%) or secondary level of education (22.7%). Majority of the participants (65.3%) were married. Retirees (28.8%) and Civil servants (26.3%) formed the majority of the participants as shown in Table 1.

**Table 1:** Socio-demographic characteristics of study participants.

	Total	T2DM	Control	*X*^2^	*P*-value
	*N* = 472, *n* (%)	N= 354, *n* (%)	*N* = 118, *n* (%)		
**Gender**			1.40	0.28	
Male	198 (41.9)	154 (43.5)	44 (37.3)		
Female	274 (58.1)	200 (56.5)	749 (62.7)		
**Occupation**			6.4	0.17	
Traders	93 (19.7)	78 (22)	15 (12.7)		
Civil Servants	133 (28.2)	93 (26.3)	40 (33.9)		
Private sectors	102 (21.6)	76 (21.5)	26 (22.0)		
Students	8 (1.7)	5 (1.4)	3 (2.5)		
Retirees/No employment	136 (28.8)	102 (28.8)	34 (28.8)		
**Educational status**			0.42	0.94	
None	138 (29.2)	103 (29.1)	35 (29.7)		
1^0^ Education	46 (9.7)	33 (9.3)	13 (11.0)		
2^0^ Education	107 (22.7)	82 (23.2)	25 (21.2)		
3^0^ Education	181 (38.3)	136 (38.4)	45 (38.1)		
**Marital status**			0.3	0.85	
Singles	11 (2.3)	9 (2.5)	2 (1.7)		
Married	310 (65.7)	231 (65.3)	79 (66.9)		
Widowed	151 (32.0)	114 (32.2)	37 (31.4)		
**Family history of DM**			14.0	0.001^*^	
Yes	163 (34.5)	139 (39.3)	24 (20.3)		
No	309 (65.5)	215 (60.7)	94 (79.7)		

* *p-value is statistically significant*.

The mean age of the T2DM patients in this study was 57.5 (±9.3) years, while the controls had a similar mean age of 57.7 ± 8.9 (*p* = 0.17). The mean age at diagnosis of DM was 54 ± 7.6 years. The mean duration of DM for all the T2DM patients was 6.5 ± 2.8 years. Table 2 below shows that T2DM patients had higher mean BMI than the controls (27.6 ± 5.0 vs. 26.2 ± 3.8). This difference was statistically significant (*t* = 2.7; *p* = 0.002). The T2DM patients also had higher mean waist circumference (90.2 ± 13.9 vs. 87.5 ± 11.0), and this difference was also statistically significant (*t* = −2.2, *p* = 0.03).

**Table 2:** Clinical characteristics of study participants.

	**T2DM patients**	**Control**	***T*-value**	***P*-value**
	***N* = 354**	***N* = 118**		
Mean age (years)	57.5 (±9.3)	57.7 (±8.9)	0.17	0.17
BMI (kg/m^2^)	27.6 (±5.0)	26.2 (±3.8)	−2.7	0.002^*^
Waist circumference (cm)	90.2 (±13.9)	87.5 (±11.0)	−2.2	0.03^*^
Weight (kg)	72.0 (±14.2)	69.1 (±12.0)	2.10	0.04^*^
Height (m)	1.62 (±0.7)	1.62 (±0.8)	−0.46	0.65
Systolic BP (mmHg)	130.4 (±18.7)	127.5 (±18.1)	−1.4	0.15
Diastolic BP (mmHg)	81.7 (±9.5)	80.2 (±9.1)	−1.5	0.13

* *P-value is statistically significant*.

### Complications of DM

The type 2 DM patients in this study had the following chronic complications of DM: DM retinopathy (49.1%), peripheral neuropathy (48.1%), DM nephropathy (13.0%), and DM foot ulcer (19.8%)

Thyroid dysfunction had a statistically significant relationship with DM nephropathy in this study as shown in the Table 3. The other chronic complications of DM did not have significant relationship with thyroid dysfunction in this study.

**Table 3:** The relationship between the presence of DM complications and thyroid dysfunction.

DM complications	Thyroid Yes, *N* = 44, *n* (%)	Dysfunction No, *N* = 310, *n* (%)	χ^2^	*p*-values
**Retinopathy**			1.37	0.26
Yes	18 (40.9)	156 (50.3)		
No	26 (59.1)	154 (49.7)		
**Nephropathy**			29.2	0.001^*^
Yes	17 (8.6)	29 (9.4)		
No	27 (6.4)	281 (90.6)		
**Peripheral neuropathy**			0.2	0.75
Yes	20 (45.5)	152 (49.0)		
No	24 (54.5)	158 (51.0)		
**DM foot ulcer**			1.2	0.32
Yes	6 (13.6)	64 (20.6)		
No	38 (86.4)	246 (79.4)		

* *p-value is statistically significant*.

### Distribution of Thyroid Status Among Type 2 DM Patients

Using a combination of TSH, fT3, and fT4 values, 12.4% of the T2DM patients were observed to have thyroid dysfunction (hypothyroidism-11.6%; or hyperthyroidism- 0.8%) as shown in the Table 4.

**Table 4:** Univariate analysis of the possible risk factors of thyroid dysfunction.

Variables	Thyroid Yes, *N* = 44, *n* (%)	Dysfunction No, *N* = 310, *n* (%)	χ^2^	*p*-values
**Gender**			20.3	0.001^*^
Male	11 (25)	121 (39.0)		
Female	33 (75)	189 (61.0)		
**Age (years)**			0.3	0.73
≥60	13 (29.5)	104 (33.5)		
<60	31 (70.5)	206 (66.5)		
**Duration of DM (years)**			7.7	0.005^*^
<5	34 (77.3)	171 (55.2)		
≥5	10 (22.7)	139 (44.8)		
**HbA1c (%)**			5.0	0.04^*^
≥7	27 (61.4)	135 (43.5)		
<7	17 (38.6)	175 (56.5)		
**Hypertension**			0.65	0.43
Yes	20 (45.5)	161 (51.9)		
No	24 (54.5)	149 (48.1)		
**Central obesity**			24.8	0.001^*^
Yes	30 (68.2)	93 (30.0)		
No	14 (31.8)	217 (70.0)		
**Nephropathy**			29.2	0.001^*^
Yes	17 (8.6)	29 (9.4)		
No	27 (6.4)	281 (90.6)		
**Retinopathy**			1.37	0.26
Yes	18 (40.9)	156 (50.3)		
No	26 (59.1)	154 (49.7)		
**Peripheral neuropathy**			0.2	0.75
Yes	20 (45.5)	152 (49.0)		
No	24 (54.5)	158 (51.0)		
**DM foot ulcer**			1.2	0.32
Yes	6 (13.6)	64 (20.6)		
No	38 (86.4)	246 (79.4)		

* *p-value is statistically significant*.

### Possible Predictors/Risk Factors of Thyroid Dysfunction

There was a significant association between the presence of thyroid dysfunction and female gender (χ^2^ = 20.3; *p* = 0.001), duration of DM >5 years (χ^2^ = 7.7, *p* = 0.005), central obesity (χ^2^ = 24.8; *p* = 0.001), nephropathy (χ^2^ = 29.2, *p* = 0.001), and HbA1c ≥7% (χ^2^ = 5.0, *p* = 0.04) as shown in Table 4.

Binary logistic regression showed that female gender (OR = 3.8, *p* = 0.002), central obesity (OR = 2.5, *p* = 0.001), DM duration >5 years (OR = 3.3, *p* = 0.012), HbA1c ≥7 (OR = 4.3, *p* = 0.025), and DM nephropathy (OR = 4.8, *p* = 0.001) were risk factors for thyroid dysfunction.

## Discussion

Females formed 58.1% of the participants. Females also constituted 56.5% of the type 2 DM patients. This female preponderance is similar to the 60.5% reported by the Diabcare Nigeria study group in 2012 in a multi-center study that assessed the profile of Nigerians with DM (43). It is also close to 57.9% reported by Okafor et al. in Enugu, Nigeria in 2012 in a study that evaluated the cardio-metabolic risk factors in Nigerians living with type 2 DM (44).

The mean age of type 2 DM patients in this study was 57.5 years. This may be due to the fact that the prevalence of type 2 DM increases with age (37, 45, 46). This reflects the pattern observed by Chinenye et al. (57.1) in a multi-center study involving DM patients (43). Ofoegbu et al. reported a mean age of 59.2 years in Enugu in a study that evaluated the body composition of Nigerians with DM (47). Okafor et al. in Enugu reported 55.7 years as the mean age of type 2 DM patients they evaluated for cardio-metabolic risk factors (44). This is lower than the figures reported from developed countries like New Zealand, and the USA (48, 49). This can be attributed to the lower life expectancy of Africans, and Nigerians in particular, compared to those of patients in the developed world. The mean age at diagnosis of type 2 DM in this study was 54 years which is similar to that observed in the UKPDS (54 years) (50), but is a bit higher than 48.3 years reported by the Diabcare Nigeria study group (43). The mean duration of DM of 6.5 years is similar to 6.7 years observed by Okafor et al. in Enugu and reflects the pattern reported in other Nigerian studies (43, 44), but is low when compared to that observed in the developed world (49). This might mean that most diabetics in Enugu and other parts of Nigeria do not survive long enough, which might be a pointer to the disease burden and quality of care available to the patients.

Diabetic retinopathy was observed in 49.1% of the type 2 DM patients in this study. This can be attributed to the increasing prevalence of DM retinopathy. This is close to 42.1% reported by Ashaye et al. (51) in a study that evaluated retinopathy among type 2 DM patients in Ibadan, Nigeria (51), but is high when compared to the 34.6% global prevalence reported by Yau et al. (52). The prevalence of peripheral neuropathy in this study was 48.1%. This is lower than 75% prevalence reported by Ugoya et al. in Jos, Nigeria (53), but is higher than 31.2% observed by Ibrahim et al. in Kano, Nigeria (54).

Diabetic nephropathy was observed in 13% of the patients with type 2 DM in this study. This is a bit lower than 16.6% observed by Ulasi et al. in Enugu, Nigeria (55) and is much lower than 72.6% reported by Onovughakpo-Sakpa et al. in Benin, Nigeria (56).

Diabetic nephropathy was the only microvascular complication of DM that has a significant relationship with the presence of thyroid dysfunction (χ^2^ = 29.2, *p* = 0.001); and had a strong association with thyroid dysfunction on logistic regression (OR = 4.8, 95%CI = 2.2–10.5). This shows that type 2 DM patients with nephropathy are 4.8 times as likely to also have thyroid dysfunction as their counterparts who do not have nephropathy. This agrees with the report of Yau et al., that hypothyroidism is a risk factor for nephropathy (57).

### The Predictors/Risk Factors of Thyroid Dysfunction in Type 2 DM

This study observed female gender, central obesity, duration of DM >5 years, and HbA1c ≥7% as independent predictors of thyroid dysfunction in type 2 DM (see Table 5).

**Table 5:** Multivariate Analysis (logistic regression) of predictors/risk factors of thyroid dysfunction in type 2 DM patients.

Variables	OR	CI (95%)	Regression coefficient	*p*-values
Female gender	3.8	1.7–8.4	1.32	0.002^*^
Duration of DM (>5 years)	3.3	1.5–7.9	−1.046	0.012^*^
HbA1c (≥7%)	4.3	2.1–8.9	0.8	0.025^*^
DM nephropathy	4.8	2.2–10.5	1.57	0.001^*^
Central obesity	2.5	1.2–5.2	−1.378	0.001^*^

* *p-value is statistically significant*.

Females who had type 2 DM were 3.8 times more likely to develop thyroid dysfunction than their male counterparts. This reflects the findings of a study from India which reported that the prevalence of thyroid disorders were more in females as compared to males (69 vs. 31%) (58). These findings are also consistent with several other studies (59–62). Thus, the prevalence of thyroid disorders in diabetic patients is influenced by female gender.

Central obesity (abnormal waist circumference) was significantly associated with thyroid dysfunction (OR = 2.5, 95%CI = 1.5–5.2, *p* = 0.001). This is similar to the report of Udenze et al. in Lagos, Nigeria who reported that waist circumference had a significant association with thyroid dysfunction (63). Biondi et al. also found associations between thyroid dysfunction and obesity in the metabolic syndrome (64). This may be as a result of the link between obesity and leptin. Leptin is known to be an important neuro-endocrine regulator of the hypothalamo-pituitary-thyroid axis by regulating TRH gene expression in the paraventricular nucleus (65). Iodine deficiency, autoimmune thyroiditis and mutations in the TSH receptor genes are some of the other hypothesis put forward to explain the association between increasing TSH, obesity and subclinical hypothyrodism in some populations (63, 66).

Elevated HbA1c (poor glycaemic control) has been shown to be strongly associated with chronic complications of DM (50, 67). This study observed that type 2 DM patients with elevated HbA1c were 4.3 times more likely to develop thyroid dysfunction than their counterparts with good glycaemic control (HbA1c<7%). This may be due the adverse effects of chronic hyperglycaemia on the hypothalamo-pituitary axis where it blunts or abolishes the nocturnal TSH peak (7). Several previous studies have categorized clinical and subclinical hypothyroidism as insulin resistant states. Bazrafshan et al. in his study (68) found a significant correlation between HbA1c levels and TSH levels which is similar to our findings. In a study by Ardekani et al. (69), HbA1c were significantly higher in patients with diabetes having thyroid disorders which is in keeping with findings from our study.

Hyperglycaemia also inhibits the peripheral deiodination of T4 to T3 by reducing the activities of thyroxine deiodinase (8). Schlienger et al. in their study (70) on the “Effect of diabetic control on the level of circulating thyroid hormones” reported that a poor diabetic control (glycosylated hemoglobin ≥12%) is associated with a low T3 syndrome as a result of impairment of T4 to T3 conversion.

This study reported that DM duration >5 years (OR = 3.3, *p* = 0.012) was a risk factor for thyroid dysfunction. Furthermore, there was a significant difference (*p* = 0.001) in the mean duration of DM between those that have thyroid dysfunction (9.5 years) and those that are euthyroid (6.0 years). This might be an indication that increasing duration of DM may be a risk factor in the prevalence of thyroid dysfunction as chronic hyperglycaemia impairs the peripheral deiodination of T4 to T3 leading to thyroid dysfunction. Which is also in keeping with findings by Telwani et al. (58) who reported that the prevalence of thyroid disorders was significantly more in diabetics with duration of diabetes = 5 years as compared to duration < 5 years (75.9 vs. 24.1%). However, study by Diez et al., found no significant relationship between presence of thyroid dysfunction and duration of diabetes (71).

## Conclusion

In conclusion, female gender, central obesity, HbA1c (≥7%), duration of DM (>5 years), and DM nephropathy were risk factors of thyroid dysfunction in type 2 DM patients in this study.

## Limitations of the Study

Assaying for thyroid hormones using the more sensitive chemilumniscent immunoassay method would have sought out more patients with thyroid dysfunction. Another limitation is the inability of the authors to do fasting blood glucose in the control group and in T2DM patients in this study. Thirdly, limitations deriving from the possible influence of metformin on thyroid function and from the use of a qualitative method to measure proteinuria deserve mention.

## Ethics Statement

Ethical approval was obtained from the University of Nigeria Health Research Ethics Committee of the UNTH Enugu before commencing the study. Written informed consent was obtained from all subjects participating in the study.

## Author Contributions

SO and IE conceived the study, participated in its design and coordination, helped to draft the manuscript, participated in the design of the study, and performed the statistical analysis. IE participated in the sequence alignment. All authors read and approved the final manuscript.

### Conflict of Interest Statement

The authors declare that the research was conducted in the absence of any commercial or financial relationships that could be construed as a potential conflict of interest.

## Abbreviations

BMI: Body Mass Index
CLIA: chemilumniscent immunoassay
DM: diabetes mellitus
fT3: free 3,5,3-triiodothyronine
fT4: free 3,5,3′,5′-tetraiodothyronine
HbA1c: glycosylated hemoglobin
IBM: International Business Machine
NHANES III: National Health and Nutritional Education Survey III
RBG: random blood glucose
SPSS: Statistical Package for the Social Sciences
T1DM: type 2 diabetes mellitus
T2DM: type 2 diabetes mellitus
TSH: thyroid stimulating hormone
UKPDS: United Kingdom Prospective Diabetes Study
UNTH: University of Nigeria Teaching Hospital.
